# Redox-Active Drug, MnTE-2-PyP^5+^, Prevents and Treats Cardiac Arrhythmias Preserving Heart Contractile Function

**DOI:** 10.1155/2020/4850697

**Published:** 2020-03-21

**Authors:** Andrezza M. Barbosa, José F. Sarmento-Neto, José E. R. Menezes Filho, Itamar C. G. Jesus, Diego S. Souza, Valério M. N. Vasconcelos, Fagner D. L. Gomes, Aline Lara, Juliana S. S. Araújo, Sandra S. Mattos, Carla M. L. Vasconcelos, Silvia Guatimosim, Jader S. Cruz, Ines Batinic-Haberle, Demetrius A. M. Araújo, Júlio S. Rebouças, Enéas R. Gomes

**Affiliations:** ^1^Department of Biotechnology, Federal University of Paraiba, Joao Pessoa, Brazil; ^2^Heart Circle, Recife, Brazil; ^3^Department of Chemistry, Federal University of Paraiba, Joao Pessoa, Brazil; ^4^Department of Physiology, Federal University of Sergipe, Aracaju, Brazil; ^5^Department of Physiology and Biophysics, Federal University of Minas Gerais, Belo Horizonte, Brazil; ^6^Department of Public Health, Federal University of Paraiba, Joao Pessoa, Brazil; ^7^Department of Biochemistry and Immunology, Federal University of Minas Gerais, Belo Horizonte, Brazil; ^8^Department of Radiation Oncology, Duke University School of Medicine, Durham, NC 27710, USA

## Abstract

**Background:**

Cardiomyopathies remain among the leading causes of death worldwide, despite all efforts and important advances in the development of cardiovascular therapeutics, demonstrating the need for new solutions. Herein, we describe the effects of the redox-active therapeutic Mn(III) *meso*-tetrakis(*N*-ethylpyridinium-2-yl)porphyrin, AEOL10113, BMX-010 (MnTE-2-PyP^5+^), on rat heart as an entry to new strategies to circumvent cardiomyopathies.

**Methods:**

Wistar rats weighing 250-300 g were used in both *in vitro* and *in vivo* experiments, to analyze intracellular Ca^2+^ dynamics, L-type Ca^2+^ currents, Ca^2+^ spark frequency, intracellular reactive oxygen species (ROS) levels, and cardiomyocyte and cardiac contractility, in control and MnTE-2-PyP^5+^-treated cells, hearts, or animals. Cells and hearts were treated with 20 *μ*M MnTE-2-PyP^5+^ and animals with 1 mg/kg, i.p. daily. Additionally, we performed electrocardiographic and echocardiographic analysis.

**Results:**

Using isolated rat cardiomyocytes, we observed that MnTE-2-PyP^5+^ reduced intracellular Ca^2+^ transient amplitude, without altering cell contractility. Whereas MnTE-2-PyP^5+^ did not alter basal ROS levels, it was efficient in modulating cardiomyocyte redox state under stress conditions; MnTE-2-PyP^5+^ reduced Ca^2+^ spark frequency and increased sarcoplasmic reticulum (SR) Ca^2+^ load. Accordingly, analysis of isolated perfused rat hearts showed that MnTE-2-PyP^5+^ preserves cardiac function, increases SR Ca^2+^ load, and reduces arrhythmia index, indicating an antiarrhythmic effect. *In vivo* experiments showed that MnTE-2-PyP^5+^ treatment increased Ca^2+^ transient, preserved cardiac ejection fraction, and reduced arrhythmia index and duration. MnTE-2-PyP^5+^ was effective both to prevent and to treat cardiac arrhythmias.

**Conclusion:**

MnTE-2-PyP^5+^ prevents and treats cardiac arrhythmias in rats. In contrast to most antiarrhythmic drugs, MnTE-2-PyP^5+^ preserves cardiac contractile function, arising, thus, as a prospective therapeutic for improvement of cardiac arrhythmia treatment.

## 1. Introduction

Therapeutic improvements, lifestyle modifications, and wider adoption of evidence-based medicine have resulted in a remarkable 30–35% decline in cardiovascular mortality [1]. However, despite all efforts and advances in developing cardiovascular therapeutics, cardiomyopathies are still a major public health problem and the main causes of death around the world [2, 3].

Since the first observation that Ca^2+^ was required for cardiac contraction and pacemaker activity, the role of Ca^2+^ as a signaling ion in the heart has been progressively dissected and better understood at molecular level, and it is clear that abnormalities in Ca^2+^ homeostasis play a pivotal role in the pathogenesis of many cardiovascular diseases, including cardiac arrhythmias [4]. Inherited gene alteration and acquired defects of multiple Ca^2+^-handling proteins can contribute to the pathogenesis of arrhythmias in different categories of heart disease [4]. However, drug therapy is available only for some of these conditions and is often only partially effective [5].

Ca^2+^ handling within cardiomyocytes is widely recognized as a potential target for the treatment of cardiac disease. Whereas the role of Ca^2+^ channels in cardiac muscle contraction has long been elucidated [6], the biophysical and genetic identities of various voltage-gated Ca^2+^ channels were disclosed [7, 8], contributing along the way to several classes of antagonists being described, which comprise now part of the formulary for the treatment of cardiac diseases including arrhythmias [4].

Accordingly, Ca^2+^ channel blockers are able to decrease the automaticity of ectopic foci in the heart and can be used in many arrhythmias [4]. Overall, it is thought that reduced L-type calcium currents (*I*_Ca,L_) result in less Ca^2+^ overload on the myocyte, reducing tendency to ectopy, which can trigger arrhythmias [4]. Additionally, cardiotonic glycosides or digitalis are positive inotropes used in clinical practice for the treatment of heart failure that also behave as endogenous ligands for Na^+^/K^+^ ATPase. An increase in intracellular Ca^2+^ content mediates their positive inotropic effect but has also been suggested as a trigger of life-threatening arrhythmias [9].

In many tissues, including the heart, reactive oxygen/nitrogen species (ROS/RNS) are often derived from mitochondria, NADPH oxidase, or uncoupled-nitric oxide synthase (NOS) and are kept under tight homeostatic control [10, 11]. In the cardiovascular system, ROS/RNS has been shown to play an important role in regulation of K^+^, Na^+^, L-type Ca^2+^ channels (in plasmatic membrane), and ryanodine receptor (RyR2) in sarcoplasmic reticulum membrane [12–14]. Mn-porphyrin-based compounds have been widely recognized as potent redox-active therapeutics, being able to modulate ROS/RNS in several animal models of oxidative stress [15–18]. Mn(III) *meso*-tetrakis(*N*-ethylpyridinium-2-yl) porphyrin (MnTE-2-PyP^5+^), also known as AEOL10113 or BMX-010, is currently under phase I/II clinical trials in Canada and the USA [15], and preclinical toxicological studies in conscious telemetered male cynomolgus monkeys showed that administration of MnTE-2-PyP^5+^ at a dose of 1 mg/kg/day led to no statistically significant changes in heart rate or arterial blood pressure [19].

The pharmacokinetic studies on MnTE-2-PyP^5+^ show a good distribution of this compound into the heart [20, 21]. Such data prompted us to investigate MnTE-2-PyP^5+^ as a redox-active experimental therapeutic for cardiomyopathy, with particular focus on reducing Ca^2+^ stress and preserving cardiac contractile function. We demonstrate that MnTE-2-PyP^5+^ exerts protective effects in rat hearts, by modulating Ca^2+^ dynamics, reducing arrhythmia score, and preserving contractile function of the heart. Additionally, MnTE-2-PyP^5+^ was effective in preventing and treating cardiac arrhythmias *in vivo*.

## 2. Methods

### 2.1. Animals

All experiments were performed using rats of both sexes (*Rattus norvegicus*, 200–250 g). Animals were maintained at the Federal University of Paraiba (UFPB), Brazil, in accordance with NIH guidelines for the care and use of animals. Experiments were performed according to approved animal protocols from the Institutional Animal Care and Use Committee at UFPB (CEUA Protocol 016/2017). All animals were euthanized by decapitation.

### 2.2. MnTE-2-PyP^5+^ Synthesis

MnTE-2-PyP^5+^ was synthesized and characterized as described elsewhere [22–25]. Concentrations of MnTE-2-PyP^5+^ stock solutions were determined spectrophotometrically (log *ε*_454nm_ = 5.14) [22–24]. Moreover, MnTE-2-PyP^5+^ is extremely stable toward demetallation, even in strong concentrated acids (e.g., 98% sulfuric acid) [26–28], or in the presence of strong chelating agents, such as EDTA [26–29]. For both *in vitro* and *in vivo* experiments, MnTE-2-PyP^5+^ was diluted in 0.9% NaCl sterile solution.

### 2.3. Cardiomyocyte Isolation and Ca^2+^ Recordings

Ventricular rat cardiomyocytes were isolated and stored until they were used as previously described [30]. Intracellular Ca^2+^ analysis was performed with Fluo-4 AM (10 *μ*M; Invitrogen, Eugene, OR)-loaded cardiomyocytes. The cells were stained for 30 min and then washed to remove the excess dye. Cells were electrically stimulated at 1 Hz to produce steady-state conditions. The images were recorded in a Zeiss LSM 510META confocal microscope. As an indicator of the SR Ca^2+^ load, 10 mM caffeine stimulation (in a Ca^2+^- and Na^+^-free solution) and the amplitude of the Ca^2+^ transient evoked were recorded [31]. Preconditioning pulses (1 Hz) were used in the cells before caffeine was applied. Ca^2+^ spark frequencies were recorded in resting ventricular myocytes. The Ca^2+^ level was reported as *F*/*F*_0_ (or as Δ*F*/*F*_0_), where *F*_0_ is the resting Ca^2+^ fluorescence.

### 2.4. ROS Recordings

Isolated cardiomyocytes were incubated with 10 *μ*M dihydroethidium (DHE, Molecular Probes, Eugene, OR) for 30 min at 37°C and were subsequently washed with an extracellular solution to remove the excess dye. Images were acquired with a Zeiss LSM 510 META confocal microscope. Images were analyzed in ImageJ software.

### 2.5. Measurement of L-Type Ca^2+^ Current

Whole-cell voltage-clamp recordings were done at 22–25°C using an EPC-9.2 patch-clamp amplifier (HEKA Electronics, Rheinland-Pfalz, Germany) as described previously [32, 33]. L-type Ca^2+^ current (*I*_Ca,L_) measurement was done using internal solution as follows (in mM): 5 NaCl, 120 CsCl, 20 TEACl, 5 EGTA, and 10 HEPES and pH 7.2 (adjusted using CsOH 1.0 M). External solution composition was as follows (in mM): 5.4 KCl, 140 NaCl, 1.8 CaCl_2_, 1 MgCl_2_, 10 HEPES, and 10 glucose and pH 7.2 (adjusted with NaOH 1.0 M). To measure the effect of MnTE-2-PyP^5+^ on *I*_Ca,L_ density, we used a holding potential of −80 mV. Next, to inactivate both voltage-gated Na^+^ channels and T-type Ca^2+^ channels, a prestep of 50 ms to −40 mV was applied. Then, the membrane potential was swapped to 0 mV for 300 ms. This protocol was used before, during, and after washing off each concentration of MnTE-2-PyP^5+^.

### 2.6. Measurement of LV Myocyte Shortening

Cellular contractility was evaluated as previously described [34]. Briefly, isolated myocytes were placed in a chamber mounted on the stage of an inverted microscope (Eclipse TS 100; Nikon, Japan). The chamber was perfused with Tyrode's solution (in mM): 150 NaCl, 5.4 KCl, 0.5 MgCl_2_, 1.8 CaCl_2_, 10 HEPES, and 10 glucose and pH set at 7.4. All experiments were performed at room temperature. Myocytes were stimulated to contract at 1 Hz with 4 ms square pulse. Shortening was measured using a video-edge detection acquisition system (IonOptix, Milton, MA, USA). Sarcomeric shortening was expressed as a percentage of diastolic LV myocyte length. Five consecutive myocyte contractions were averaged before analysis. Cell shortening, maximal rates of contraction and relaxation, and times to 10% contraction and relaxation were determined for all groups.

### 2.7. Atrial Contractility

The left atrium was mounted in an organ chamber and maintained in modified Krebs–Henseleit solution (KHS) containing (in mM) 120 NaCl, 5.4 KCl, 1.2 MgCl_2_, 1.25 CaCl_2_, 11 glucose, 27 NaHCO_3_, and 2 NaH_2_PO_4_ (pH 7.4), oxygenated with carbogen mixture (95% O_2_ and 5% CO_2_) and maintained at 29 ± 0.1°C. The atrium was electrically stimulated (1 Hz, 80 V, 1.5 ms, SD9 stimulator, Grass). Tissue was placed under 5 mN tension, and an isometric force transducer (HP FTA 10-1 Sunborn) was used to record the contraction force. After 30 min of stabilization, MnTE-2-PyP^5+^ was added cumulatively to the bath (1, 3, 10, 30, 100, and 300 *μ*M).

### 2.8. Sarcoplasmic Reticular Ca^2+^ Load

The heart was quickly removed, placed in a Krebs–Henseleit solution continuously bubbled with 95% 0_2_ and 5% CO_2_, and the left atrium was dissected. The left atrium was tied with an isometric force transducer (Grass FT03) which was mounted vertically on a micromanipulator. Stimulation (STIMULATOR SD9 GRASS) was with pulses of 0.5 ms duration at a suprathreshold voltage, and frequency of stimulation was 1 Hz for a 30 min equilibration period. Steady level and postrest contractions following 20 s of pause in stimulation were observed in caffeine-treated (10 mM) or caffeine-treated containing 20 *μ*M MnTE-2-PyP^5+^. Measurements were made on the last contraction before the pause and on the first contraction after the rest interval.

### 2.9. Langendorff Preparation-Perfused Hearts

Animals were euthanized 10–15 min after intraperitoneal injection of 1000 IU heparin/kg. The heart was dissected and perfused through a 1.0 ± 0.3 cm aortic stump with Krebs–Henseleit solution (KHS) containing (in mM) NaCl, 120; KCl, 5.4; MgCl_2_, 1.2; CaCl_2_, 1.25; glucose, 11; NaHCO_3_, 27; and NaH_2_PO_4_, 2 (pH 7.4). The perfusion fluid was maintained at 34 ± 1°C with a pressure of 10 ml/min in constant oxygenation (5% CO_2_ and 95% O_2_). Electrical activity was recorded utilizing three platinum electrodes (Ag/AgCl, in NaCl 1 M electrolytic solution) placed inside the chamber close to the heart for sensing electrical signals. Hearts were perfused for an initial 20 min period with KHS. After equilibration, the hearts were perfused for 12 min with KHS, 12 min with KHS+20 *μ*M MnTE-2-PyP^5+^, and last 30 min period with KHS. The high-calcium model was used to determine cardiac arrhythmias. Therefore, after a 20 min stabilization, hearts were perfused for 25 min with normal perfusion KHS at 34°C, started 25 min with high calcium (HC) (3.3 mM) and 25 min with HC+20 *μ*M MnTE-2-PyP^5+^.

### 2.10. *In Vitro* Arrhythmia

*In vitro* arrhythmia was determined in an isolated heart as described previously [35]. Hearts were subjected to perfusion with KHS containing 1.25 mM of calcium (control group) at 34°C during 20 min. After stabilization, the hearts were subjected to perfusion with 3.3 mM high calcium (HC group) or with high calcium in the presence of 20 *μ*M MnTE-2-PyP^5+^ during 25 min. Arrhythmia scores were determinate as previously described [36]. Therefore, to quantify the arrhythmias, 25 min of experiment was divided into 3 min intervals and the arrhythmia scores were added at the end.

### 2.11. Measurement of Left Ventricular Pressure

Left intraventricular pressure was measured using a water-filled balloon introduced into the cavity of the left ventricle with a constant diastolic pressure of 15 mmHg by adjusting the volume of the balloon, connected to a pressure transducer (FE221, Bridge Amp, ADInstruments, Australia) coupled to an amplifier (PowerLab 8/35, ADInstruments). Ventricular pressures were processed using a dedicated software (LabChart 8 Pro, ADInstruments).

### 2.12. *In Vivo* MnTE-2-PyP^5+^ Safety

To test the *in vivo* safety of MnTE-2-PyP^5+^, animals were randomized into two groups: (1) control 0.9% saline (1 ml/kg/day, i.p.) and (2) MnTE-2-PyP^5+^ (1 mg/kg/day, i.p.), which were treated for 15 days. The dose regimen for MnTE-2-PyP^5+^ administration was chosen based on rat model experiments carried out previously by our group [17, 37, 38].

### 2.13. *In Vivo* ECG Measurements

The animals were anesthetized with ketamine (80 mg/kg, i.p.); surface ECG measurements were conducted using subdermal electrodes placed in the DII lead arrangement connected to a cardioscope, amplified and digitalized (PowerLab 4/35 ADInstruments, USA). ECG signals were recorded for 15 min, then animals received an injection of caffeine (120 mg/kg, i.p.) and epinephrine (2 mg/kg, i.p.). Data were analyzed in LabChart 8 (ADInstruments, USA) and arrhythmic score measured as described previously [36].

### 2.14. *In Vivo* Arrhythmia Susceptibility

The animals were injected with dexamethasone (4 mg/kg, i.p.) for 7 days to predispose to arrhythmia. In the prevention protocol, animals received MnTE-2-PyP^5+^ (1 mg/kg/day, i.p.) during the 7 days of dexamethasone administration. In the treatment protocol, the animals received only dexamethasone until day 5 and MnTE-2-PyP^5+^ (1 mg/kg/day, i.p.) and dexamethasone (4 mg/kg, i.p.) in days 6 and 7. Rats were randomized into four experimental groups: 1: control; 2: dexamethasone; 3: dexamethasone+MnP (treatment); and 4: dexamethasone+MnP (prevention).

### 2.15. M-Mode Echocardiography

Cardiac function under noninvasive conditions was assessed by two-dimensional guided M-mode echocardiography of halothane-anesthetized mice as previously described [39]. Briefly, animals were positioned on supine position with front paws wide open and trichotomized. Transthoracic echocardiography was performed using a SonoSite M-Turbo Ultrasound System B (USA) equipped with a 14 MHz linear transducer.

### 2.16. Statistical Analysis

Data are presented as mean ± SEM. Sample comparisons were performed using Student's *t*-test for two-group analysis or one-way ANOVA followed by post hoc analysis for multiple comparisons. In all statistical tests, a *p* < 0.05 was used as a measure of statistical significance.

## 3. Results

### 3.1. MnTE-2-PyP^5+^ Reduces Ca^2+^ Signaling Preserving Cardiomyocyte Contractility

**Figure 1:**
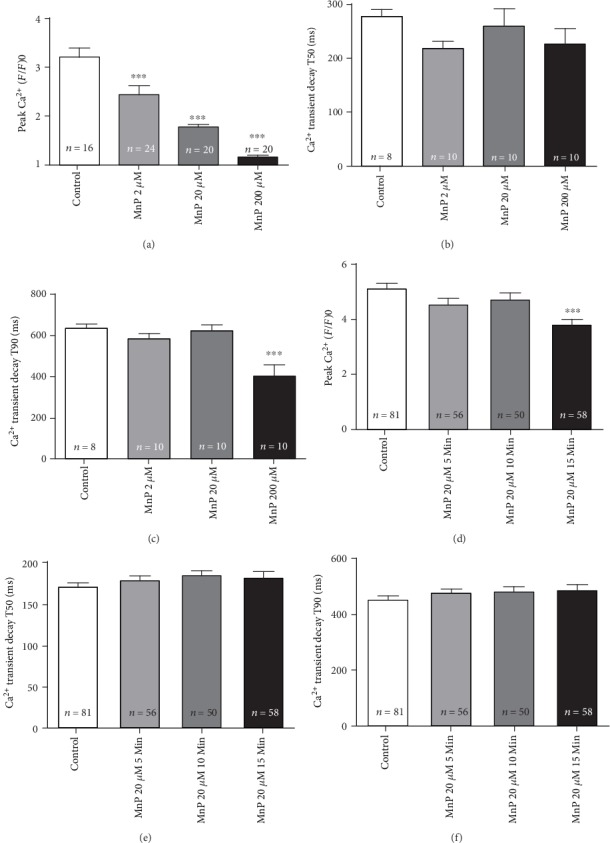
MnTE-2-PyP^5+^ reduces Ca^2+^ transients in cardiac myocytes. Concentration effect of MnTE-2-PyP^5+^ on Ca^2+^ transients. (a) Significant reduction in peak Ca^2+^ transient amplitude in isolated cardiomyocytes treated with MnTE-2-PyP^5+^. Ca^2+^ transient kinetics of decay in ms for (b) T50 or (c) T90. (d, e) Time course effect of MnTE-2-PyP^5+^ 20 *μ*M on Ca^2+^ transients. (d) Significant reduction in peak Ca^2+^ transient amplitude in MnTE-2-PyP^5+^-treated cardiomyocytes for 15 min. (e, f) Ca^2+^ transient kinetics of decay in ms for (e) T50 or (f) T90; *n* = at least 10 cells per animal and 3 animals per group. ^∗^*p* ≤ 0.05; ^∗∗^*p* ≤ 0.01; ^∗∗∗^*p* ≤ 0.001. MnP = MnTE-2-PyP^5+^.

To investigate whether MnTE-2-PyP^5+^ modulates Ca^2+^ signaling in the heart, we performed Ca^2+^ transient analysis in isolated cardiomyocytes loaded with Fluo-4/AM (5 *μ*M), incubated for 90 min with crescent concentrations of MnTE-2-PyP^5+^ (2-200 *μ*M), and observed a significant reduction in peak Ca^2+^ transient in a concentration-dependent manner (Figure 1(a)). However, the kinetics of Ca^2+^ decay was altered only for T90 at 200 *μ*M concentration (Figures 1(b) and 1(c)). Considering that 20 *μ*M MnTE-2-PyP^5+^ concentration reduced approximately by 50% the peak Ca^2+^ transient, we chose this concentration to continue the experiments. To further understand the MnTE-2-PyP^5+^ effect on cardiac myocytes, we performed a time course analysis of Ca^2+^ transient from 5 to 15 min. Once again, MnTE-2-PyP^5+^ promoted a significant decrease in peak Ca^2+^ transient, observed after 15 min of incubation (Figure 1(d)), without causing alterations in the kinetics of Ca^2+^ decay (Figures 1(e) and 1(f)). Additionally, we observed that MnTE-2-PyP^5+^ did not change the basal Fluo-4 fluorescence (Supplementary videos [Supplementary-material supplementary-material-1]).

**Figure 2:**
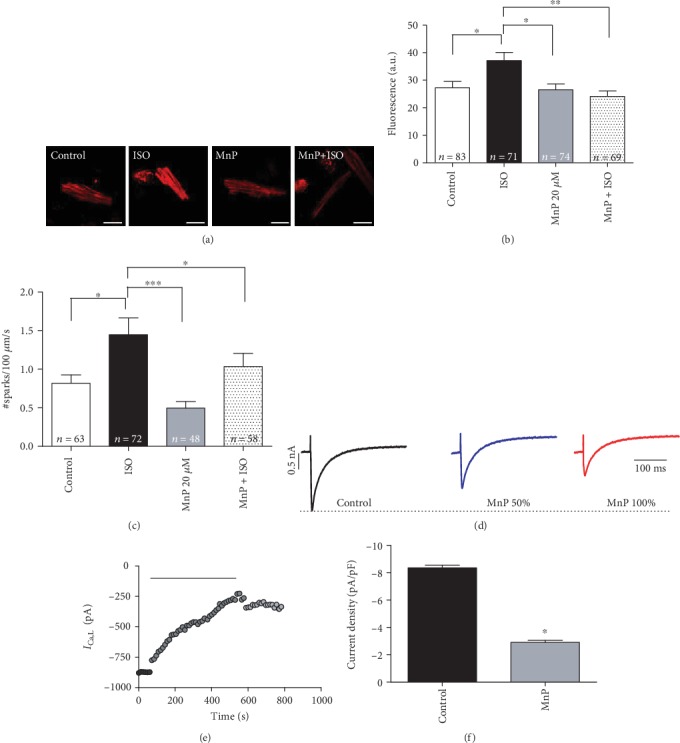
MnTE-2-PyP^5+^ reduces Ca^2+^ currents independent of antioxidant activity. (a) Representative images of DHE-stained cells. Scale bar = 10 *μ*m. (b) Bar graph showing that MnTE-2-PyP^5+^ does not change basal ROS levels but prevents ISO-induced ROS increase. (c) MnTE-2-PyP^5+^ prevents ISO-induced increase in Ca^2+^ spark frequency. (d) Representative traces of *I*_Ca,L_. (e) Time course of *I*_Ca,L_ current. (f) Bar graph showing *I*_Ca,L_ density reduction by MnTE-2-PyP^5+^. *n* = at least 10 cells per animal and 3 animals per group. ^∗^*p* ≤ 0.05; ^∗∗^*p* ≤ 0.01; ^∗∗∗^*p* ≤ 0.001. MnP = MnTE-2-PyP^5+^.

Considering that MnTE-2-PyP^5+^ is a superoxide dismutase (SOD) mimetic (currently recognized as a broad redox modulator) [17, 18], we used dihydroethidium (DHE), a fluorescent, cell-permeable, reactive oxygen species (ROS) indicator, to evaluate MnTE-2-PyP^5+^ effect on cardiomyocyte ROS levels. MnTE-2-PyP^5+^ did not change the basal levels of ROS. However, as its well-known that MnTE-2-PyP^5+^, physiologically, has antioxidant activity under oxidative stress conditions, we used isoproterenol (ISO) as a cell stressor. As expected, ISO induced an increase in DHE fluorescence that was prevented by MnTE-2-PyP^5+^ (Figures 2(a) and 2(b)), indicating that MnTE-2-PyP^5+^ is efficient in modulating cardiomyocyte redox state under stress conditions. Nevertheless, as MnTE-2-PyP^5+^ did not change basal ROS levels in cardiomyocytes, this result suggests that the observed reduction in Ca^2+^ transient induced by MnTE-2-PyP^5+^ is likely independent of its antioxidant effect.

As L-type Ca^2+^ channels (LTCC) and ryanodine receptors (RyR) are critical for normal Ca^2+^ signaling, we investigated the MnTE-2-PyP^5+^ effects on L-type Ca^2+^ current (*I*_Ca,L_) using whole-cell voltage-clamp recordings. Figures 2(d)–2(f) show that MnTE-2-PyP^5+^ induced a significant reduction in *I*_Ca,L_ current density, compatible with the reduction in the peak Ca^2+^ transient. Additionally, we measured Ca^2+^ spark frequency, and interestingly, MnTE-2-PyP^5+^ reduced basal spark frequency (Figure 2(c)) and prevented the increase in Ca^2+^ spark frequency induced by ISO (Figure 2(c)).

**Figure 3:**
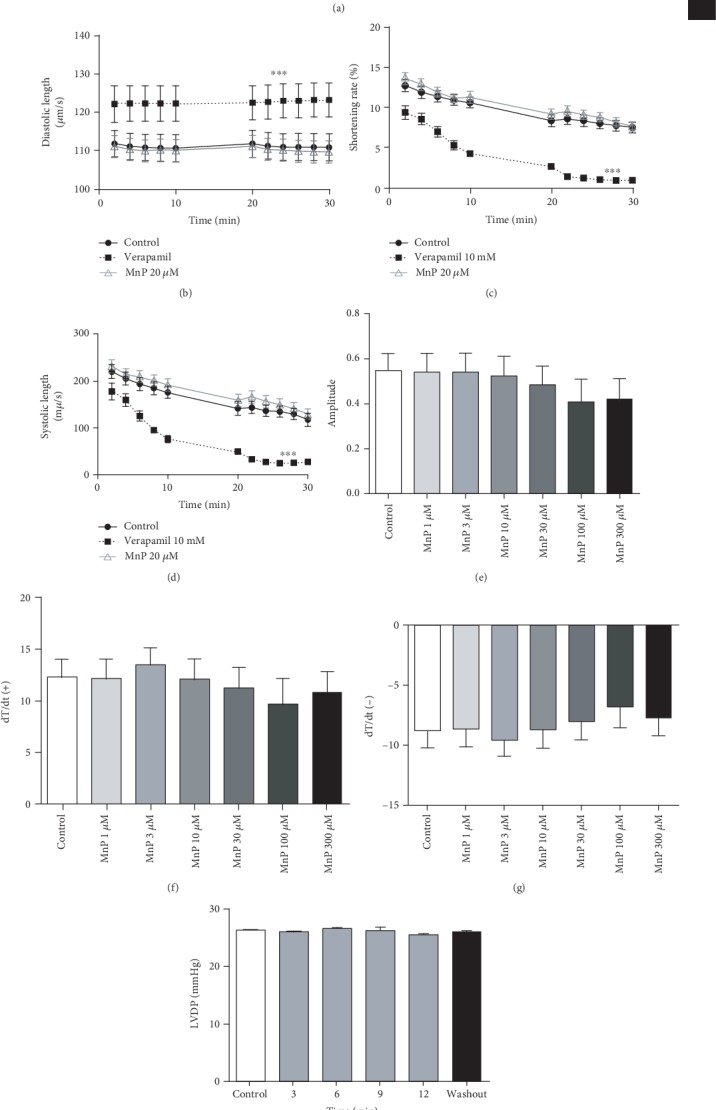
MnTE-2-PyP^5+^ preserves cardiomyocyte and atria contractility. (a) Representative traces of isolated ventricular cardiomyocyte contractility. Time course of (b) diastolic length, (c) shortening rate, and (d) systolic length in MnTE-2-PyP^5+^-treated cardiomyocytes. Concentration effect of MnTE-2-PyP^5+^ on isolated perfused hearts' (e) amplitude of contraction, (f) dT/dt(+), and (g) dT/dt(-). (h) Bar graph showing time course effect of MnTE-2-PyP^5+^ on LVDP. LVDP = left ventricular diastolic pressure. (b–d) *n* = at least 10 cells per animal and 5 animals per group. (e–h) *n* = 5 animals per group. ^∗^*p* ≤ 0.05; ^∗∗^*p* ≤ 0.01; ^∗∗∗^*p* ≤ 0.001. MnP = MnTE-2-PyP^5+^.

As we observed all these alterations in pivotal components of excitation-contraction coupling (ECC) and considering the importance of Ca^2+^ ion for cellular contraction, we next analyzed the MnTE-2-PyP^5+^ effects on cardiomyocyte contractility. Remarkably, despite the reduction in *I*_Ca,L_ and in peak Ca^2+^ transient, no changes in fractional shortening, systolic length, or diastolic length of MnTE-2-PyP^5+^-treated cardiomyocytes were observed, which is in direct contrast to the LTCC blocker verapamil-treated group (Figures 3(a)–3(d)). This result is noteworthy, because it indicates that although MnTE-2-PyP^5+^ decreases Ca^2+^ transient, it preserves cardiomyocyte contractility.

### 3.2. MnTE-2-PyP^5+^ Preserves Heart Contractility Reducing Arrhythmia Index

Based on effects observed in isolated cardiomyocytes, we decided to investigate MnTE-2-PyP^5+^ actions on heart contractility. First, using isolated left atria preparation, it was observed that MnTE-2-PyP^5+^ did not evoke alterations in contraction force, dT/dt(+), and dT/dt(-) (Figures 3(e)–3(g)), corroborating the data obtained in isolated cardiomyocytes and indicating that MnTE-2-PyP^5+^ preserves cardiomyocyte contractility, also in tissue analysis. Furthermore, in Langendorff-perfused hearts, MnTE-2-PyP^5+^ did not change the left ventricular developed pressure (LVDP) (Figure 3(h)). Additionally, we verified that systole, diastole, and cardiac cycle duration were not modified by MnTE-2-PyP^5+^ (Supplementary Fig. [Supplementary-material supplementary-material-1]). Overall, these data show that although MnTE-2-PyP^5+^ acutely reduces Ca^2+^ signaling in isolated cardiomyocytes, it preserves heart contractility.

As we observed alterations in Ca^2+^ spark frequency in isolated cardiomyocytes, we also tested if MnTE-2-PyP^5+^ altered SR Ca^2+^ content in isolated atria. Consistent with isolated cell data, MnTE-2-PyP^5+^ increased the SR Ca^2+^ load in approximately 66%, which helps to explain the maintenance of the cardiac contractility (Supplementary Figs. [Supplementary-material supplementary-material-1]).

**Figure 4:**
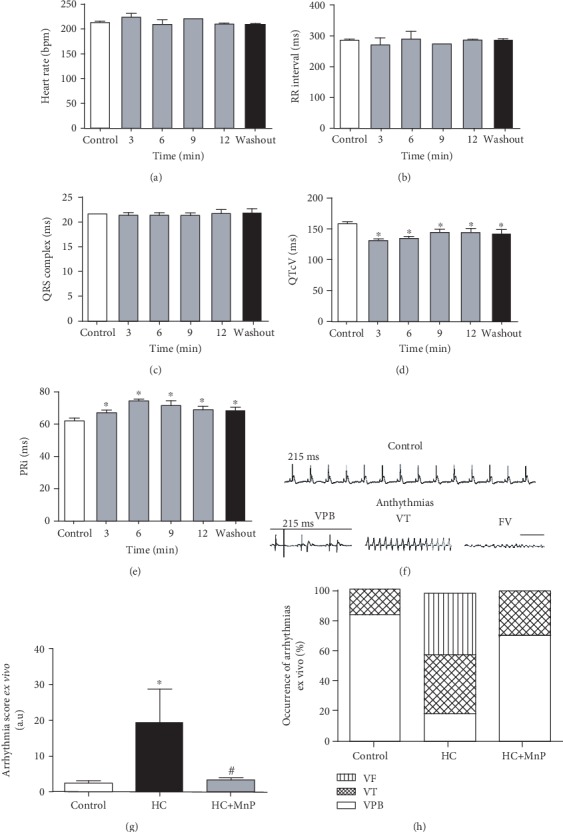
MnTE-2-PyP^5+^ preserves cardiac electrical activity and prevents high calcium-induced arrhythmias. Bar graphs showing (a) significant increase in PR interval, (b) RR interval, (c) QRS complex duration, (d) significant reduction in adjusted QT duration, and (e) heart rate. (f) Representative electrocardiographic traces. Bar graphs showing MnTE-2-PyP^5+^ preventive effect on (g) arrhythmia score and (h) severity. *n* = 5 animals per group. ^∗^*p* ≤ 0.05; ^∗∗^*p* ≤ 0.01; ^∗∗∗^*p* ≤ 0.001. ^#^*p* ≤ 0.05 compared to control. Mn = MnTE-2-PyP^5+^.

Considering the involvement of Ca^2+^ ions in triggering cardiac arrhythmias, we used Langendorff-perfused hearts to test whether MnTE-2-PyP^5+^ could alter the electrical activity of the heart. Electrocardiographic (ECG) recordings were used to analyze ECG intervals and segments. The results showed that heart rate and QRS complex length were not modified by MnTE-2-PyP^5+^ (Figures 4(a)–4(c)). However, QTcV was significantly shortened and PRi increased (Figures 4(d) and 4(e)). Additionally, to investigate if MnTE-2-PyP^5+^ could target cardiac arrhythmias, isolated hearts were perfused with high calcium (HC) to induce cardiac arrhythmias.  Figure 4(f) shows normal ECG traces in control situation (top panel) and induced arrhythmias in HC-perfused heart (bottom panel). As shown in Figure 4(g), HC perfusion significantly increased the arrhythmia scores and MnTE-2-PyP^5+^ decreased HC-induced cardiac arrhythmias. Furthermore, most of the arrhythmias evidenced in control situation were of lower severity, such as ventricular premature beats (VPB, Figure 4(h)). In contrast, HC-perfused hearts presented ventricular fibrillation (VF), the most severe type of arrhythmia. Remarkably, MnTE-2-PyP^5+^ prevented the incidence of the most severe arrhythmia events (Figure 4(h)).

### 3.3. MnTE-2-PyP^5+^ Increases Ca^2+^ Transient and Preserves Heart Function *In Vivo*

**Figure 5:**
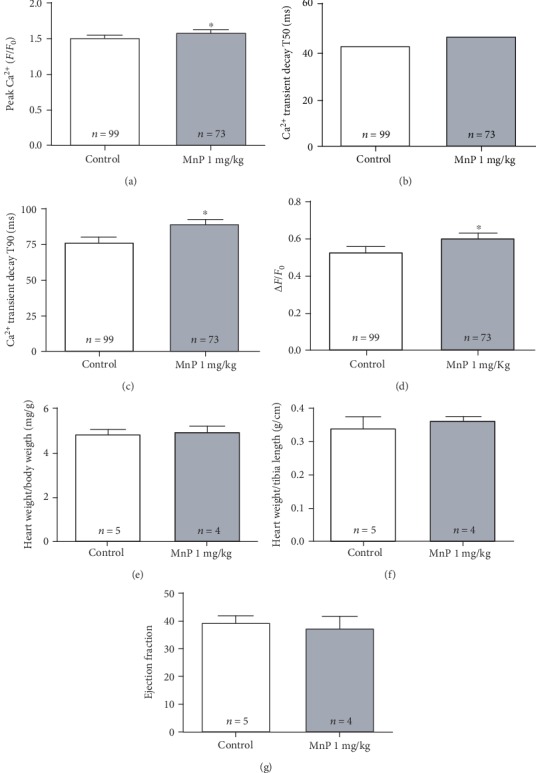
MnTE-2-PyP^5+^ (1 mg/kg/day, i.p., 15 days) increases Ca^2+^ transients *in vivo* and preserves cardiac mass and function. (a–c) Effect of MnTE-2-PyP^5+^ on Ca^2+^ transients. (a) Significant increase in peak Ca^2+^ transient amplitude in cardiomyocytes from animals treated with MnTE-2-PyP^5+^. Ca^2+^ transient kinetics of decay in ms for (b) T50 or (c) T90. (d) SR load is increased in cells from MnTE-2-PyP^5+^-treated animals. MnTE-2-PyP^5+^ preserves cardiac mass when measured by (e) heart weight/body weight or (f) heart weight/tibia length. (g) Bar graph of echocardiographic measurement of ejection fraction showing that MnTE-2-PyP^5+^ preserves systolic function *in vivo*. (a–d) *n* = number of cells (at least 5 animals per group). (e–g) *n* = number of animals. ^∗^*p* ≤ 0.05; ^∗∗^*p* ≤ 0.01; ^∗∗∗^*p* ≤ 0.001. MnP = MnTE-2-PyP^5+^. VF = ventricular fibrillation; VT = ventricular tachycardia; VPB = ventricular premature beat; HC = high calcium; MnP = MnTE-2-PyP^5+^. MnTE-2-PyP^5+^ 20 *μ*M per 25 minutes was used in all experiments.

Based on our *in vitro* results, we visualized MnTE-2-PyP^5+^ as a potential lead for therapeutic approaches for some cardiac arrhythmias. Thus, we designed *in vivo* experiments in rats to investigate the effect of MnTE-2-PyP^5+^ on the heart. Animals were treated daily with 1 mg/kg MnTE-2-PyP^5+^ (i.p. injections) for 15 days. First, we tested MnTE-2-PyP^5+^ effects on Ca^2+^ transient. Cardiomyocytes from MnTE-2-PyP^5+^-treated rats presented increased peak Ca^2+^ transient and T90 time for Ca^2+^ decay, different from what we observed in acutely treated isolated myocytes (Figures 5(a)–5(c)). Additionally, as we verified an increase in SR load *in vitro*, we decided to investigate the SR load in cardiomyocytes isolated from treated animals. In agreement with *in vitro* experiments, MnTE-2-PyP^5+^ treatment increased SR Ca^2+^ load by approximately 14% (Figure 5(d)).

Additionally, heart weight/body weight (HW/BW) and heart weight/tibia length (HW/TL) ratios were evaluated as markers of cardiac hypertrophy in MnTE-2-PyP^5+^-treated animals. As shown in Figures 5(e) and 5(f), these parameters did not differ from those of the control group. Next, by echocardiographic analysis, we verified that MnTE-2-PyP^5+^-treated animals had no difference in ejection fraction (EF) when compared to control (Figure 5(g)), indicating that MnTE-2-PyP^5+^ preserves cardiac contractility *in vivo*. Taken together, these results indicate that MnTE-2-PyP^5+^ administered at 1 mg/kg/day i.p. does not alter normal heart function *in vivo*.

### 3.4. MnTE-2-PyP^5+^ Effectively Prevents and Treats Cardiac Arrhythmias *In Vivo*

**Figure 6:**
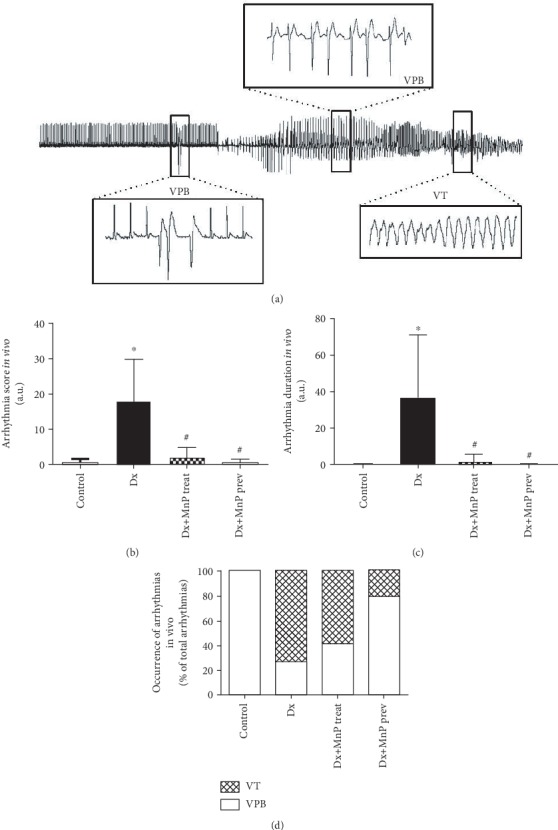
MnTE-2-PyP^5+^ effectively prevents and treats cardiac arrhythmias. (a) Representative images of electrocardiogram records, cardiac arrhythmias are highlighted. Bar graphs showing that MnTE-2-PyP^5+^ reduces (b) arrhythmia score and (c) duration. (d) Bar graph showing protective effect of MnTE-2-PyP^5+^ on severity of arrhythmias. *n* = 5 animals per group. Dx = dexamethasone (4 mg/kg, i.p. 7 days); Dx+MnP treat = dexamethasone+MnP treatment (MnTE-2-PyP^5+^, 1 mg/kg/day, i.p., during the last 2 days of Dx); Dx+MnP = dexamethasone+MnP prevention (MnTE-2-PyP^5+^, 1 mg/kg/day, i.p., during the 7 days of Dx). ^∗^*p* ≤ 0.05; ^∗∗^*p* ≤ 0.01; ^∗∗∗^*p* ≤ 0.001. ^#^*p* ≤ 0.05 compared to control. VT = ventricular tachycardia; VPB = ventricular premature beat. Mn = MnTE-2-PyP^5+^.

Considering the robust effects of MnTE-2-PyP^5+^ in preventing cardiac arrhythmias in isolated hearts, we assessed its antiarrhythmic property *in vivo*. By using a dexamethasone-induced arrhythmia model, we tested both preventive and therapeutic actions of MnTE-2-PyP^5+^.  Figure 6(a) shows representative ECG traces along with the type of the recorded arrhythmias: isolated VPB, sustained VPB, and VT. Remarkably, in both prevention and treatment protocols, MnTE-2-PyP^5+^ was completely effective to reduce both arrhythmia score and duration (Figures 6(b) and 6(c)), restoring the control profile. When we analyzed the severity of ventricular arrhythmias, we observed that MnTE-2-PyP^5+^ prevented the occurrence of ventricular tachycardia (VT) (Figure 6(d)). However, although MnTE-2-PyP^5+^ reduced the duration of arrhythmias, it was not able to reverse the relative occurrence of VT (Figure 6(d)).

**Figure 7:**
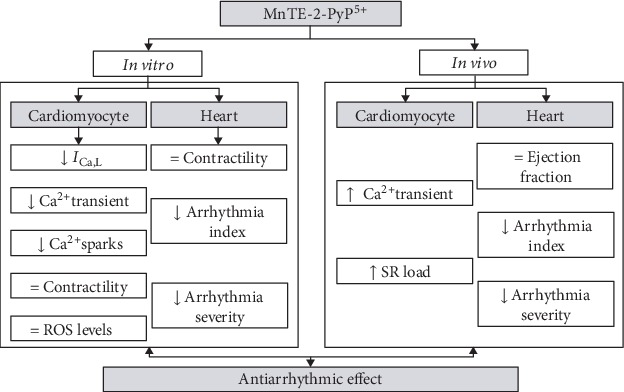
Schematic summarization of the main effects of MnTE-2-PyP^5+^ both in vitro and in vivo and the consequent antiarrhythmic effect. *I*_Ca,L_ = L-type calcium current; ROS = reactive oxygen species; SR = sarcoplasmic reticulum.

To better view the multiple actions of MnTE-2-PyP^5+^, Figure 7 presents a schematic summary of the main effects of MnTE-2-PyP^5+^ both *in vitro* and *in vivo*.

To better view the multiple actions of MnTE-2-PyP^5+^, Figure 7 presents a schematic summary of the main effects of MnTE-2-PyP^5+^ both *in vitro* and *in vivo*.

## 4. Discussion

Cardiac arrhythmias are important causes of sudden death; thus, proper treatment of these conditions is of utmost importance. Indeed, over the last two decades, there has been a great deal of progress in arrhythmia management. However, most antiarrhythmic drugs proved ineffective or dangerous in patients with ventricular arrhythmias [40], demonstrating the need for new therapeutic strategies.

Although the mainstay of treatment for catecholaminergic polymorphic ventricular tachycardia (CPVT) has been *β*-blockade, there has also been early evidence that blocking *I*_Ca,L_ with the LTCC blocker verapamil prevents ventricular arrhythmias [41]. Overall, it is thought that reduced *I*_Ca,L_ results in less Ca^2+^ overload of the myocyte, reducing predisposition to ectopy that can trigger arrhythmias [4]. In agreement with these findings, our study shows that acute administration of MnTE-2-PyP^5+^ to isolated cardiomyocytes reduced the peak Ca^2+^ transient in these cells, in association with reduced *I*_Ca,L_. Additionally, acute administration of MnTE-2-PyP^5+^ in isolated hearts resulted in reduction in arrhythmia index, severity, and duration of arrhythmias, demonstrating, for the first time, that MnTE-2-PyP^5+^ represents a new lead molecule for the treatment of cardiac arrhythmias.

Additionally, while *in vitro* acute use of MnTE-2-PyP^5+^ reduced Ca^2+^ transient in cardiomyocytes, *in vivo* 15-day use of MnTE-2-PyP^5+^ in healthy rats did not change Ca^2+^ transient in cardiomyocytes. Although these results may at first seem inconsistent, when we consider that MnTE-2-PyP^5+^ reduced Ca^2+^ spark rate and increased SR load, chronically, these two effects combined may account for the final increased Ca^2+^ transient observed *in vivo*. Additionally, as we and others [15, 17, 18] demonstrated that under stress conditions MnTE-2-PyP^5+^ prevents oxidative stress, this effect can also contribute, chronically, to cellular restoration of basal transient kinetics. Accordingly, Almeida et al. [42] demonstrated that aldosterone-treated cardiomyocytes presented increased *I*_Ca,L_ and Ca^2+^ transient and that Angiotensin-(1–7) restored basal *I*_Ca,L_ albeit with a great increase in Ca^2+^ transient. Further investigation demonstrated that this alteration in Ca^2+^ transient was caused by reduction in Ca^2+^ spark frequency and consequent increased SR load. MnTE-2-PyP^5+^ apparently works via a similar mechanism by improving Ca^2+^ transient in the long term. In addition, the systemic antioxidant effect of MnTE-2-PyP^5+^ must be considered in the cardiovascular health *in vivo*.

By reducing peripheral vasoconstriction and LV afterload, calcium channel blockers were thought to have a potential role in the management of chronic heart failure (HF). However, first-generation dihydropyridine and nondihydropyridine calcium channel blockers also have myocardial depressant activity [43]. Several clinical trials have demonstrated either no clinical benefit or even worse outcomes in patients with HF treated with these drugs [44–48]. Despite their greater selectivity for calcium channels in vascular smooth muscle cells, second-generation calcium channel blockers, dihydropyridine derivatives such as amlodipine and felodipine, have failed to demonstrate any functional or survival benefit in patients with HF [49–53]. Together, these data show that although calcium channel blockers have an important role in the management of cardiac arrhythmias, they are of limited use, especially in patients with HF.

Although the use of calcium channel blockers for arrhythmia treatment is often plagued by myocardial depressant activity, here, we demonstrated that MnTE-2-PyP^5+^ prevents and treats cardiac arrhythmias while preserving contractility at both cardiomyocyte and heart levels. These combined effects respond to a large gap in arrhythmia treatments, especially in patients with HF.

It is worth noting that our study demonstrates that MnTE-2-PyP^5+^ preserves cardiomyocyte and heart contractility and exerts antiarrhythmic effects both *in vitro* and *in vivo*, representing, thus, a potentially new strategy to treat cardiac arrhythmias in patients with contractile dysfunctions. In addition, although reduction in peak calcium transients is usually related to reduction in cardiac contractility, modulation of proteins involved in calcium handling or contractile machinery can alter this relationship. In this way, Vanzelli el al [54]. demonstrated that heart failure mice treated with carvedilol had an improvement in cardiac fractional shortening instead of no alterations in peak calcium transients. Although we did not analyze these mechanisms directly, we speculate that MnTE-2-PyP^5+^ effect may be somewhat related to the mechanism described by Vanzelli el al [54].; further investigations are obviously needed to disclose the actual MnTE-2-PyP^5+^ mechanism(s) of action.

Finally, it is important to highlight that the use of Mn porphyrin-based SOD mimics for cardiovascular treatments is in its infancy. A first report [55] was very recently published on the ability of an analogous Mn porphyrin, MnTnBuOE-2-PyP^5+^ (BMX-001) [28], to suppress aortic valve sclerosis in mice and human models. We showed herein that the prototypical Mn-porphyrin MnTE-2-PyP^5+^ represents a simple, promising redox-active therapeutic for preventing and treating cardiac arrhythmias, preserving heart contractile function. Taken together, the data strengthen the therapeutic potential of Mn porphyrins in a quite unexplored field of cardiac applications.

## Data Availability

Part of this manuscript is under patent registration. Because of that the authors do not present a data availability statement.

## Supplementary Materials

Supplementary 1 Supplementary figure 1: (A-C) duration time for systole, diastole, and cardiac cycle, calculated in electrocardiograms recorded in isolated hearts, and (D) representative traces of the left atrium contraction. Steady level and postrest contractions following 20 s of pause in stimulation were observed in caffeine-treated (10 mM) or caffeine-treated containing 20 *μ*M MnTE-2-PyP^5+^.

Supplementary 2 Supplementary figure 1: (E) the bar graph of contraction force average for each group. Measurements were made on the last contraction before the pause and on the first contraction after the rest interval. Supplementary videos 1-4: representative videos of Fluo-4 AM (10 *μ*M; Invitrogen, Eugene, OR)-loaded cardiomyocytes, under field stimulation (1 Hz), recorded in a Zeiss Axio Observer A1 fluorescent microscope.
