# Supplementary material for: Redox-Active Drug, MnTE-2-PyP5+, Prevents and Treats Cardiac Arrhythmias Preserving Heart Contractile Function
Source: Oxid Med Cell Longev. 2020 Mar 21;2020:4850697. doi: 10.1155/2020/4850697 (PMC7115175; doi:10.1155/2020/4850697)
Supplement: Supplementary 1 — Supplementary figure 1: (A-C) duration time for systole, diastole, and cardiac cycle, calculated in electrocardiograms recorded in isolated hearts, and (D) representative traces of the left atrium contraction. Steady level and postrest contractions following 20 s of pause in stimulation were observed in caffeine-treated (10 mM) or caffeine-treated containing 20 μM MnTE-2-PyP5+. [file 4850697.f1.pdf]

Supplementary Figure 1

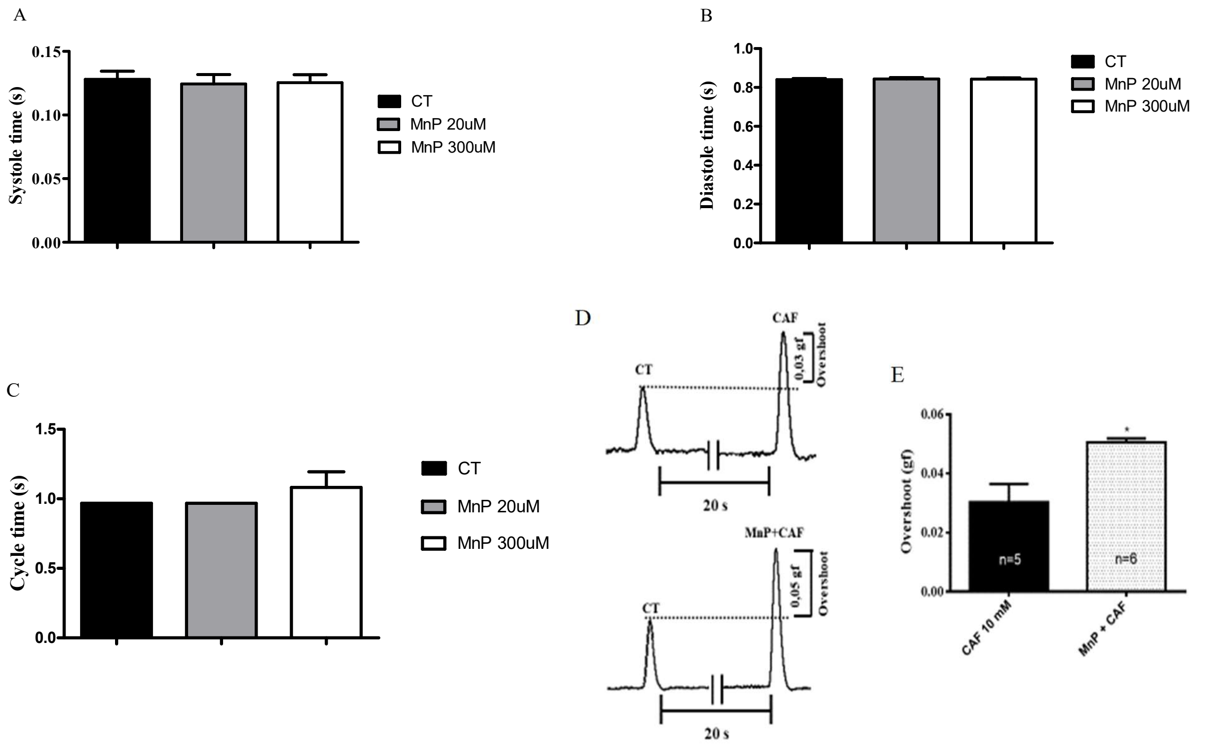

Figure S1: MnTE-2-PyP^{5+} preserves cardiac cycle phases time and increases SR load in rat hearts. A-C: Concentration effect of MnTE-2-PyP^{5+} on cardiac cycle phases time; A: Systolic time is not altered by MnTE-2-PyP^{5+}; B: Diastolic time is not altered by MnTE-2-PyP^{5+}; C: Cardiac cycle time is not altered by MnTE-2-PyP^{5+}; D: Representative traces of atria contractility, before and after caffeine stimulation, in the presence (botton) or absence (top) of MnTE-2-PyP^{5+}; E: Bar graph showing increased contractility after caffeine stimulation in MnTE-2-PyP^{5+} incubated atria. n= at least 5 hearts per group. * $p \leq 0.05$.
